# Concatenation of molecular docking and molecular simulation of BACE-1, γ-secretase targeted ligands: in pursuit of Alzheimer’s treatment

**DOI:** 10.1080/07853890.2021.2009124

**Published:** 2021-12-10

**Authors:** Nasimudeen R. Jabir, Md. Tabish Rehman, Khadeejah Alsolami, Shazi Shakil, Torki A. Zughaibi, Raed F. Alserihi, Mohd. Shahnawaz Khan, Mohamed F. AlAjmi, Shams Tabrez

**Affiliations:** a Department of Biochemistry, Centre for Research and Development, PRIST University, Thanjavur, India; b Department of Pharmacognosy, College of Pharmacy, King Saud University, Riyadh, Saudi Arabia; c Department of Pharmacology and Toxicology, College of Pharmacy, Taif University, Taif, Saudi Arabia; d King Fahd Medical Research Center, King Abdulaziz University, Jeddah, Saudi Arabia; e Department of Medical Laboratory Technology, Faculty of Applied Medical Sciences, King Abdulaziz University, Jeddah, Saudi Arabia; f Center of Excellence in Genomic Medicine Research (CEGMR), King Abdulaziz University, Jeddah, Saudi Arabia; g 3D Bioprinting Unit, Center of Innovation in Personalized Medicine, King Abdulaziz University, Jeddah, Saudi Arabia; h Department of Biochemistry, College of Sciences, King Saud University, Riyadh, Saudi Arabia

**Keywords:** Alzheimer’s disease, drug development, enzyme targets, molecular docking, multi-targeted ligands

## Abstract

**Introduction:**

Alzheimer's disease (AD), the most predominant cause of dementia, has evolved tremendously with an escalating frequency, mainly affecting the elderly population. An effective means of delaying, preventing, or treating AD is yet to be achieved. The failure rate of dementia drug trials has been relatively higher than in other disease-related clinical trials. Hence, multi-targeted therapeutic approaches are gaining attention in pharmacological developments.

**Aims:**

As an extension of our earlier reports, we have performed docking and molecular dynamic (MD) simulation studies for the same 13 potential ligands against beta-site APP cleaving enzyme 1 (BACE-1) and γ-secretase as a therapeutic target for AD. The *In-silico* screening of these ligands as potential inhibitors of BACE-1 and **γ-**secretase was performed using AutoDock enabled PyRx v-0.8. The protein-ligand interactions were analyzed in Discovery Studio 2020 (BIOVIA). The stability of the most promising ligand against BACE-1 and **γ-**secretase was evaluated by MD simulation using Desmond-2018 (Schrodinger, LLC, NY, USA).

**Results:**

The computational screening revealed that the docking energy values for each of the ligands against both the target enzymes were in the range of −7.0 to −10.1 kcal/mol. Among the 13 ligands, 8 (55E, 6Z2, 6Z5, BRW, F1B, GVP, IQ6, and X37) showed binding energies of ≤−8 kcal/mol against BACE-1 and γ-secretase. For the selected enzyme targets, BACE-1 and γ-secretase, 6Z5 displayed the lowest binding energy of −10.1 and −9.8 kcal/mol, respectively. The MD simulation study confirmed the stability of BACE-6Z5 and γ-secretase-6Z5 complexes and highlighted the formation of a stable complex between 6Z5 and target enzymes.

**Conclusion:**

The virtual screening, molecular docking, and molecular dynamics simulation studies revealed the potential of these multi-enzyme targeted ligands. Among the studied ligands, 6Z5 seems to have the best binding potential and forms a stable complex with BACE-1 and γ-secretase. We recommend the synthesis of 6Z5 for future *in-vitro* and *in-vivo* studies.

## Introduction

Alzheimer's disease (AD) is a multifactorial neurodegenerative condition represented by progressive memory deficits and cognitive decline [1,2]. It is the most predominant cause of dementia, pathologically defined by neuronal loss and combined aggregation of β-amyloid and hyper-phosphorylated tau protein [3,4]. AD has evolved tremendously and is the 6th leading cause of death in the United States, affecting the elderly population largely [5]. The next few years are expected to see a rise in AD burden as there is no effective means of delaying, preventing, or treating this disease as yet [6]. Furthermore, the failure rate of dementia drug trials (99.6%) has also been a bit higher than trials against other diseases [7].

Although the pathological features of AD are well characterized, the specific mechanisms leading to AD development and progression remain to be understood [8,9]. The modulation of amyloid metabolic cascade resulting deposition of β-amyloid and neurofibrillary tangles (NFTs) is the widely accepted hypothesis of AD pathophysiology [3,4]. The accumulation of these proteins triggers neuroinflammation, oxidative stress, and mitochondrial damage, ultimately leading to the progressive loss of neuronal structure and function [10,11]. Deficits in the cholinergic system within the nucleus basalis of Meyner have been the earliest and most studied molecular events that characterize AD pathophysiology [12–14].

Enzymes are promising therapeutic targets as AD pathology involves more than 200 enzymes/proteins [15,16]. Several reports suggested the role of β- and γ-secretase enzymes in AD pathophysiology [17–19]. They participate in the metabolism of amyloid precursor protein (APP) that forms amyloid plaques. This study chose the same ligands we reported in our earlier studies to predict its possible binding to BACE-1 and **γ-**secretase before going to the synthesis procedure [20,21]. Molecular docking is the commonly used computational tool to predict the most stable conformation of ligand in the active site of a particular target by calculating Gibbs free energy where the most negative score indicates the best stable and potent complex [22]. In this study, we performed docking and molecular dynamics simulation considering BACE-1 and **γ-**secretase as the target proteins and utilized the same set of ligands as reported earlier by our group using AutoDock-Vina and Desmond-2018 (Schrodinger, LLC, NY, USA). The results from this study are expected to lead to the synthesis of a novel compound or multiple compounds that could offer hope against AD treatment via multi-enzyme targeting.

## Materials and methods

### Experimental process

The workflow of this study included the selection and preparations of protein targets and ligands, molecular docking, and molecular dynamic (MD) simulation. DrugBank and RCSB-PDB databases (http://www.rcsb.org/pdb/) were used to identify and download the 3D structure of target enzymes and ligands. PyRx-v0.8 [23] using Autodock-Vina [24] with the Lamarckian genetic algorithm was used to generate target ligand binding affinities. Molecular interactions study was carried out using the programs Discovery Studio 2020 (BIOVIA)

### Preparation of enzyme targets

The selection of multi-targeted anti-AD enzymes was made after an exhaustive review of scientific literature. We utilized the available data from the DrugBank for the selection of enzyme targets viz. BACE-1 and **γ-**secretase. The crystal structure of BACE-1 (PDB ID: 1M4H) and **γ-**secretase (PDB ID: 6IYC) were downloaded from the RCSB protein databank in pdb format. The selection of PBD structures was made according to previous reports [25,26]. 1M4H and 6IYC have shown a reasonable resolution at 2.10 and 2.60 Å, respectively. The preparation and refining of BACE-1 and **γ-**secretase, including removal of native ligand and water molecules, assigning hydrogen polarities, calculating Gasteiger charges to protein structures, were carried out by a protein model tool in Discovery Studio 2020 (BIOVIA). Energy minimization and geometry optimization of proteins’ structures were performed using an in-built tool in PyRx-v0.8.

### Preparation of ligands

Based on our earlier study, a group of top 13 best-scored ligands that showed potential effect against multiple enzyme targets, were selected [20,21]. All the ligands were previously predicted to cross the BBB and showed gastrointestinal permeability. The library of selected ligands include 6-bromoindirubin-3′-oxime **(PDB ligand ID: BRW);** (4∼)-3-cyclopropyl-4,7,7-trimethyl-4-phenyl-2,6,8,9-tetrahydropyrazolo[3,4-b]quinolin-5-one **(PDB ligand ID: 6VK);** 5,5-dimethyl-7-[(1 ∼ {S})-4-oxidanyl-1 ∼ {H}-inden-1-yl]-2-phenylazanyl-pyrrolo[2,3d]pyrimidin-6-one **(PDB ligand ID: 6Z5);** N-(2-ethoxyethyl)-N-{(2S)-2-hydroxy-3-[(2R)-6-hydroxy-4-oxo-3,4-dihydro-1′H-spiro[chromene-2,3′-piperidin]-1′-yl]propyl}-2,6-dimethylbenzenesulfonamide **(PDB ligand ID: SMH);** 4-(4-tert-butylbenzyl)-1-(7H-pyrrolo[2,3-d]pyrimidin-4-yl)piperidin-4-aminium **(PDB ligand ID: X37);** 4-(4-hydroxy-3-methylphenyl)-6-phenylpyrimidin-2(5H)-one **(PDB ligand ID: 55E);** 4-(2-methoxyphenyl)-3,7,7-trimethyl-1,6,7,8-tetrahydro-5H-pyrazolo[3,4-b]quinolin-5-one **(PDB ligand ID: 65A);** 6-chloro-N-cyclohexyl-4-(1H-pyrrolo[2,3-b]pyridin-3-yl)pyridin-2-amine **(PDB ligand ID: IQ6);** (4 ∼ {S})-4-ethyl-7,7-dimethyl-4-phenyl-2,6,8,9-tetrahydropyrazolo[3,4-b]quinolin-5-one (**PDB ligand ID: 6VL);** (4 ∼ {S})-3-(2,2-dimethylpropyl)-4,7,7-trimethyl-4-phenyl-2,6,8,9-tetrahydropyrazolo[3,4-b]quinolin-5-one **(PDB ligand ID: 6VM);** (3 ∼ {Z})-5-ethanoyl-3-[[(1-methylpiperidin-4-yl)amino]-phenyl-methylidene]-1 ∼ {H}-indol-2-one **(PDB ligand ID: F1B);** 7-[(1 ∼ {S})-1-(4-fluorophenyl)ethyl]-5,5-dimethyl-2-(pyridin-3-ylamino)pyrrolo[2,3-d]pyrimidin-6-one **(PDB ligand ID: 6Z2)** and 4-(4-chlorophenyl)-4-[4-(1h-pyrazol-4-yl)phenyl]piperidine **(PDB ligand ID: GVP)**. All the structures were downloaded as sdf format and converted to Autodock suitable pdbqt format in PyRx-v0.8. All the ligands were energy minimized using Universal Forcefield (UFF) of PyRx-v0.8. Subsequently, the molecular and physiochemical properties, such as absorption, distribution, metabolism, and excretion of the compounds were retrieved from the available literature.

### Molecular docking

The molecular docking analysis was performed using the PyRx-v0.8 virtual screening tool coupled with AutoDock-Vina, employing the Lamarckian genetic algorithm [27–30]. All the 13 ligands were individually docked with the selected target enzymes as separate docking runs. We used a blind docking approach to predict the binding modes and docking energies, as reported previously, with the same set of ligands on different targets [21,31]. The effectiveness and reproducibility of blind docking have also been suggested as a comparatively easy method of molecular screening [32]. The grid box selection allowed the ligand to move freely within the assigned values in the X, Y, and Z planes. The 1M4H grid box dimensions were set as 104 × 108 × 89 Å having a centre at −6.65 × 44.33 × 15.60 Å, and the grid box dimensions of the grid box for 6IYC were set as 99 × 82 × 121 Å centered at 172.84 × 175.61 × 187.71 Å. Ligand’s state variables in the Lamarckian genetic algorithm including initial positions, orientations, and torsions were set randomly. All rotatable torsions were released during docking.

The results were clustered according to the root-mean-square deviation (RMSD) criterion, and this study selected the ligands with 0 RMSD modes. The docking was performed with the “exhaustiveness” set to 8. All other docking parameters were set to the default values of the software.

### Protein-ligand interactions

The best poses for each “protein-ligand complex” were generated using Discovery Studio 2020 (BIOVIA) at the end of docking. Amino acid residues involved in essential interactions and other significant contacts that clamp the ligand within the active crevice were elucidated by the “show 2D diagram”.

### Molecular dynamic simulation

The stability and dynamics of protein-ligand (BACE-1-6Z5 and γ-secretase-6Z5) complexes were evaluated by performing molecular dynamics simulation using “Desmond-2018 (Schrodinger, LLC, NY, USA)”, as reported earlier [33,34]. Briefly, the best pose of the complexes was placed in an orthorhombic box for simulation. The boundaries of the box were at least 10 Å away from the protein-ligand complex. The simulation box was solvated with TIP3P water molecules along with proper counter ions to neutralize the system. Furthermore, 0.15 M NaCl was added to mimic the physiological conditions. Optimized parameters for liquid simulation (OPLS3e) forcefield were employed to minimize the system’s energy by performing 2000 iterations with convergence criteria of 1 kcal/mol/Å. Molecular dynamics simulation was performed for 50 ns at 298 K temperature and 1 bar pressure. Nose-Hoover Chain thermostat and Martyna-Tobias-Klein barostat were utilized to maintain the temperature and pressure, respectively [35,36]. A time step of 2 fs was set, and the energies and structures were recorded at every ten ps. The binding affinity of 6Z5 for BACE-1 and γ-secretase was determined using the below-mentioned equation [37].

$$\Delta G=\;-RT\;lnK_{d}$$

where Δ*G*, *K*_d_, R, and *T* were docking free energy, binding affinity, the universal gas constant, and temperature, respectively.

## Results and discussion

The crystallographic structures of the studied enzyme targets (BACE-1 and γ-secretase) were visualized in Discovery Studio 2020 (BIOVIA). The binding pocket of BACE-1 contains proteolytic aspartic acid residues, flexible flap, and 10 seconds loop near the S3 pocket [38]. The open conformation in the active state of BACE-1 allows the substrate to enter easily and shows a significant displacement accompanied by a conformational change in the flap [39]. On the other hand, the X-ray crystal structures of human γ-secretase revealed a membrane-embedded protease complex containing two transmembrane aspartates in the active site with presenilin as the catalytic component [40].

**Figure 1.**
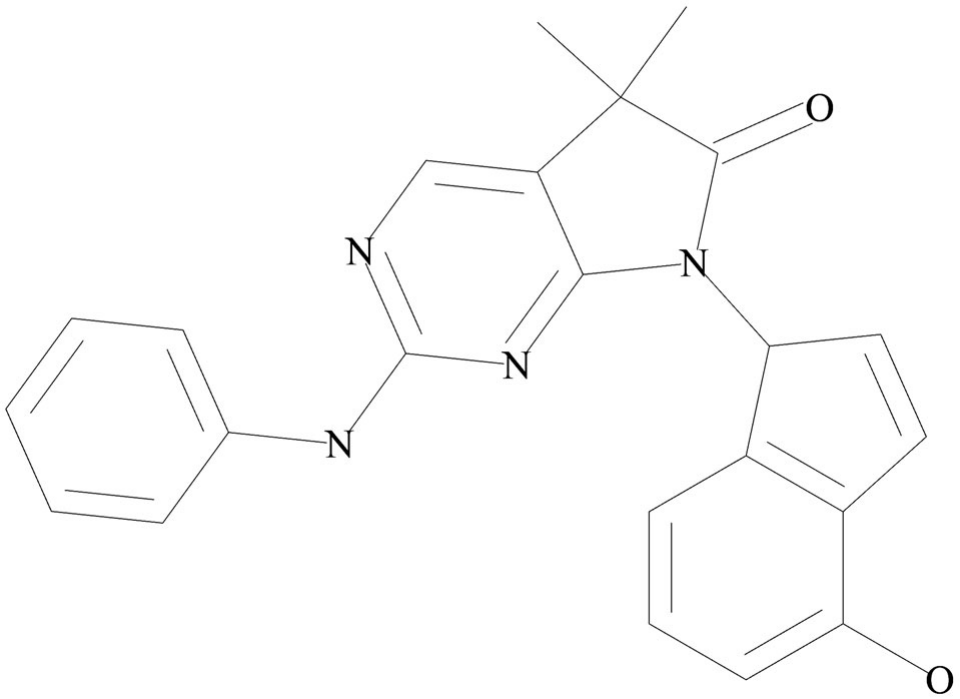
Molecular structure of the ligand “6Z5”.

**Figure 2.**
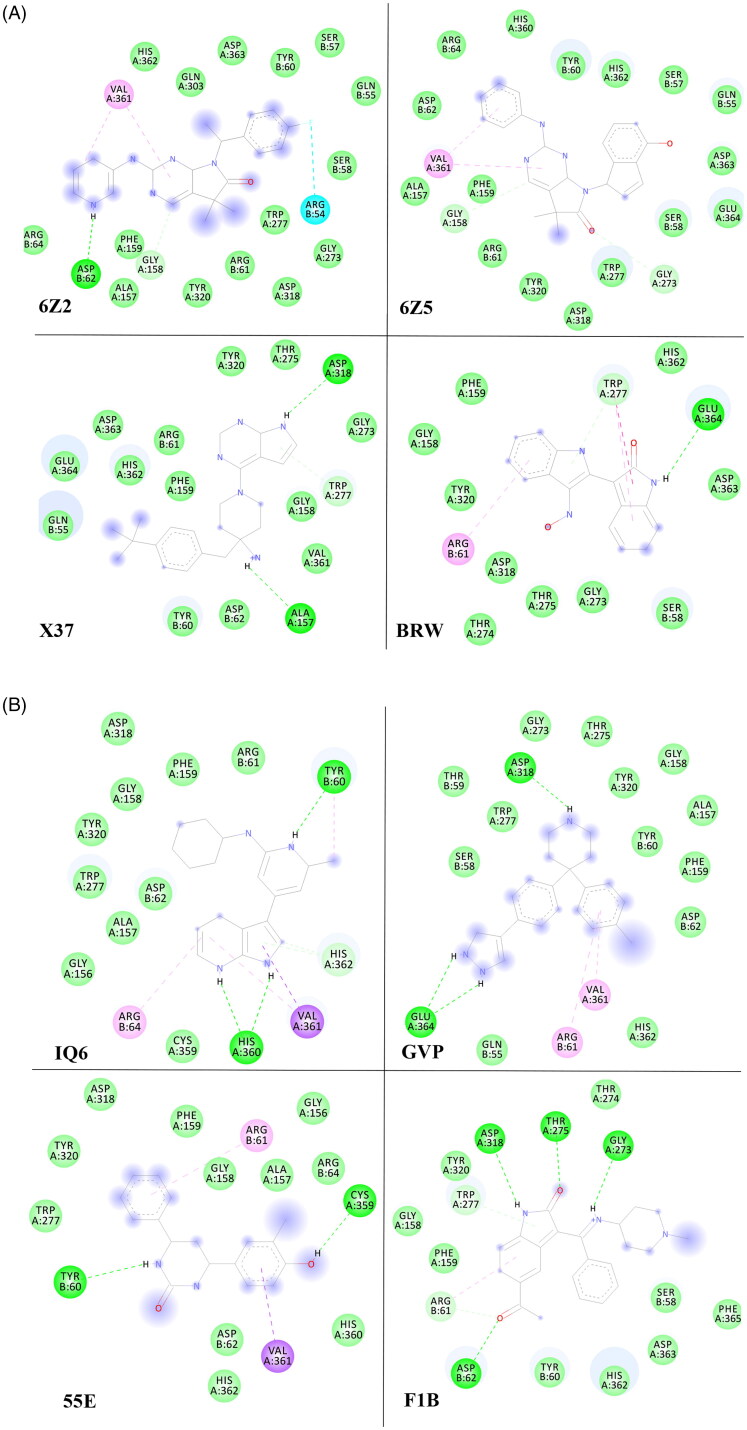
(A) The docked complexes of enzyme target BACE-1 with ligand molecules 6Z2, 6Z5, X37, and BRW. (B) The docked complexes of the enzyme target BACE-1 with ligand molecules IQ6, GVP, 55E, and F1B.

**Figure 3.**
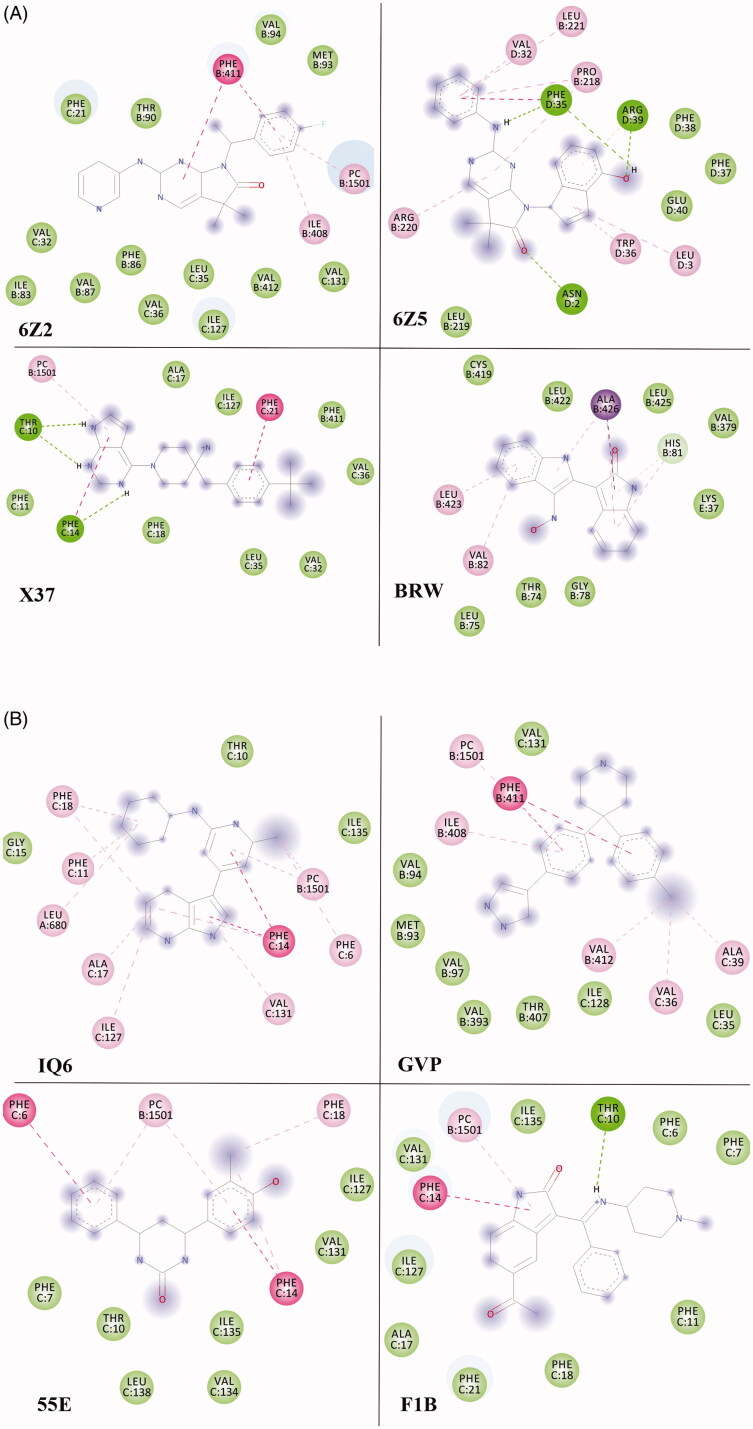
(A) The docked complexes of enzyme target γ-secretase with ligand molecules 6Z2, 6Z5, X37, and BRW. (B) The docked complexes of the enzyme target γ-secretase with ligand IQ6, GVP, 55E, and F1B.

The current study predicted the binding modes and affinities of the previously reported 13 ligands as promising multi-targeted ligand molecules against AD. The results of the predicted binding affinity of each ligand with both the protein targets are presented in Table 1. The docking energy values for the aforementioned ligands were found to be in the range of −7.0 to −10.1 kcal/mol for both the enzyme targets, BACE-1 and γ-secretase. The individual lower and upper values of binding affinity with all the studied ligands were: −8.5 to −10.1 and −7.9 to −9.8 kcal/mol, for BACE-1 and γ-secretase, respectively (Table 1). Among the 13 studied ligands, eight ligands (55E, 6Z2, 6Z5, BRW, F1B, GVP, IQ6, and X37) showed the binding affinities of ≤ −8.0 kcal/mol with both the enzymes. The ligand 6Z5 showed the lowest binding free energy of −10.1 and −9.8 kcal/mol against BACE-1 and γ-secretase, respectively. The lowest binding energy of ligand 6Z5 indicates its best possible inhibitory activity with BACE-1 and γ-secretase. The molecular structure of 6Z5 is depicted in Figure 1. Ligand-protein interactions were analyzed for each of the docking hits. Figure 2(A,B) illustrate the docked complexes of enzyme target BACE-1 with ligand molecules 6Z2, 6Z5, X37, BRW, IQ6, GVP, 55E, and F1B; while Figure 3(A,B) illustrate the complexes of **γ-**secretase with the same.

**Table 1.** Binding energies of ligands with BACE1 and γ-secretase.

No	Ligand ID	Name of the ligands	Binding energies (kcal/mol)	
			BACE1	γ-secretase
1	55E	4-(4-hydroxy-3-methylphenyl)-6-phenylpyrimidin-2(5H)-one	−8.7	−8.7
2	65A	4-(2-methoxyphenyl)-3,7,7-trimethyl-1,6,7,8-tetrahydro-5H-pyrazolo[3,4-b]quinolin-5-one	−8.9	−8.7
3	6VK	(4 ∼ {S})-3-cyclopropyl-4,7,7-trimethyl-4-phenyl-2,6,8,9-tetrahydropyrazolo[3,4-b]quinolin-5-one	−9.2	−8.6
4	6VL	(4 ∼ {S})-4-ethyl-7,7-dimethyl-4-phenyl-2,6,8,9-tetrahydropyrazolo[3,4-b]quinolin-5-one	−7.0	−8.1
5	6VM	(4 ∼ {S})-3-(2,2-dimethylpropyl)-4,7,7-trimethyl-4-phenyl-2,6,8,9-tetrahydropyrazolo[3,4-b]quinolin-5-one	−9.0	−8.2
6	6Z2	7-[(1 ∼ {S})-1-(4-fluorophenyl)ethyl]-5,5-dimethyl-2-(pyridin-3-ylamino)pyrrolo[2,3-d]pyrimidin-6-one	−9.7	−8.8
7	6Z5	5,5-dimethyl-7-[(1 ∼ {S})-4-oxidanyl-1 ∼ {H}-inden-1-yl]-2-phenylazanyl-pyrrolo[2,3-d]pyrimidin-6-one	−10.1	−9.8
8	BRW	6-bromoindirubin-3′-oxime	−8.7	−8.9
9	F1B	(3 ∼ {Z})-5-ethanoyl-3-[[(1-methylpiperidin-4-yl)amino]-phenyl-methylidene]-1 ∼ {H}-indol-2-one	−9.3	−9.4
10	GVP	4-(4-Chlorophenyl)-4-[4-(1H-pyrazol-4-YL)phenyl]piperidine	−8.6	−9.0
11	IQ6	6-chloro-N-cyclohexyl-4-(1H-pyrrolo[2,3-b]pyridin-3-yl)pyridin-2-amine	−8.9	−9.2
12	SMH	N-(2-ethoxyethyl)-N-{(2S)-2-hydroxy-3-[(2R)-6-hydroxy-4-oxo-3,4-dihydro-1′H-spiro[chromene-2,3′-piperidin]- 1′-yl]propyl}-2,6-dimethylbenzenesulfonamide	−8.5	−7.9
13	X37	4-(4-tert-butylbenzyl)-1-(7H-pyrrolo[2,3-d]pyrimidin-4-yl)piperidin-4-aminium	−9.1	−9.0
14	66F	N-{3-[(5R)-3-amino-2,5-dimethyl-1,1-dioxido-5,6-dihydro-2H-1,2,4-thiadiazin-5-yl]-4-fluorophenyl}-5-fluoropyridine- 2-carboxamide (Verubecestat, known BACE1 inhibitor)	−9.1	–
15	ESF	(2S)-2-hydroxy-3-methyl-N-[(2S)-1-[[(5S)-3-methyl-4-oxo-2,5-dihydro-1H-3-benzazepin-5-yl]amino]-1-oxopropan- 2-yl]butanamide (Semagacestat, known γ-secretase inhibitor)	–	−8.1

**Table 2.** Interaction parameters of different ligands with BACE1.

Ligands	Hydrogen bonding	Halogen bond	Pi-Sigma	Pi-Pi	Pi-Alkyl	van der Waals’ interaction
55E	Tyr 60, Cys 359	–	Val 361		Arg 61	Asp 62, Arg 64, Gly 156, Ala 157, Gly 158, Phe 159, Trp 277, Asp 318, Tyr 320, His 360, His 362
6Z2	Asp 62	Arg 54	–	–	Val 361	Gln 55, Ser 57, Ser 58, Tyr 60, Arg 61, Arg 64, Ala 157, Phe 159, Gly 273, Trp 277, Gln 303, Asp 318, Tyr 320, His 362, Asp 363
BRW	Glu 364	–	–	Arg 61	–	Ser 58, Gly 158, Phe 159, Gly 273, Thr 274, Thr 275, Asp 318, Tyr 320, His 362, Asp 363
F1B	Asp 62, Gly 273, Thr 275, Asp 318	–	–	–	–	Ser 58, Tyr 60, Gly 158, Phe 159, Thr 274, Tyr 320, His 362, Asp 363, Phe 365
GVP	Asp 318, *Glu 364	–	–	–	Arg 61, Val 361	Gln 55, Ser 58, Thr 59, Tyr 60, Asp 62, Ala 157, Gly 158, Phe 159, Gly 273, Thr 275, Trp 320, His 362
IQ6	Tyr 60, *His 360	–	Val 361	–	Arg 64	Arg 61, Asp 62, Gly 156, Ala 157, Gly 158, Phe 159, Trp 277, Asp 318, Cys 359
X37	Ala 157, Asp 318	–	–	–	–	Gln 55, Tyr 60, Arg 61, Asp 62, Gly 158, Phe 159, Gly 273, Thr 275, Tyr 320, His 362, Asp 363, Gln 364, Val 361

*Occurrence of two hydrogen bonds.

**Table 3.** Interaction parameters of different ligands with γ-secretase.

Ligands	Hydrogen bonding	Pi-Sigma	Pi-Pi	Pi-Alkyl	van der Waals’ interaction
55E	–	–	Phe 6	Phe 18	Phe 7, Thr 10, Ile 127, Val 131, Val 134, Ile 135, Leu 138
6Z2	–	–	Phe 411	Ile 408	Phe 21, Val 32, Leu 35, Val 36, Ile 83, Phe 86, Val 87, Thr 90, Met 93, Val 94, Ile 127, Val 131, Val 412
BRW	–	Ala 426	–	Val 82, Leu 423	Lys 37, Thr 74, Leu 75, Gly 78, Val 379, Cys 419, Leu 422, Leu 425
6Z5	Asn 2, *Phe 35, Arg 39		–	Leu 3, Val 32, Trp 36, Arg 220, Leu 221	Phe 37, Phe 38, Glu 40, Leu 319
F1B	Thr 10	–	Phe 14		Phe 6, Phe 7, phe 11, Ala 17, Phe 18, Phe 21, Ile 127, Val 131, Ile 135
GVP	–		Phe 411	Val 36, Ala 39, Ile 408, Val 412	Leu 35, Met 93, Val 94, Val 97, Ile 128, Val 131, Thr 393, Thr 407
IQ6	–	–	Phe 14	Phe 6, Phe 11, Ala 17, Phe 18, Ile 127, Val 131, Leu 680	Thr 10, Gly 15, Ile 135
X37	*Thr 10, Phe 14	Pro105	Phe 2	–	Phe 11, Ala 17, Phe 18, Val 32, Leu 35, Val 36, Ile 127, Phe 411

*Occurrence of two hydrogen bonds.

Ligand 6Z2 displayed one H-bonding with BACE-1 at Asp62 and π–π stacked and π-alkyl bonding at Phe411 and Ile408, respectively with **γ-**secretase (Tables 2 and 3). On the other hand, the ligand 6Z5 showed three H-bonding interactions with **γ-**secretase (Asn2, Phe35, and Arg39) and a π-alkyl interaction (Val361) along with van der Waals’ interactions with BACE-1. The ligand 55E displayed H-bonding interaction with BACE-1 (Tyr60 and Cys359), whereas it showed π-π interactions with **γ-**secretase (Phe6 and Phe14). The ligand BRW showed H-bonding interaction with BACE-1 at Glu364 whereas π-sigma and π-π interactions with **γ-**secretase (Ala426). Moreover, the ligand F1B showed H-bonding interaction with BACE-1 (Asp62, Gly273, Thr275, and Asp318) and **γ-**secretase (Thr10). Similarly, the ligand GVP showed H-bonding interaction with BACE-1 (Asp318 and Glu364), whereas it showed π-π interactions with **γ-**secretase at Phe411. The ligand IQ6 showed H-bonding interaction with BACE-1 (His360 and Tyr60), and π-π interaction was observed with **γ-**secretase (Phe14). The ligand X37 showed H-bonding interaction with BACE-1 (Ala157 and Asp318), and γ**-**secretase (Thr10 and Phe14). All of these ligands displayed acceptable pharmacokinetic properties as per their ADME evaluations [20,41]. Moreover, these ligands were also predicted to cross the blood-brain barrier by the boiled egg method; a positive feature for the future design of drugs for Alzheimer’s disease treatment.

Based on the molecular docking results, 8 out of the 13 studied ligands (55E, 6Z2, 6Z5, BRW, F1B, GVP, IQ6, and X37) showed binding energy values of ≤ −8 kcal/mol with BACE-1 (Table 1). The ligand 6Z5 showed the lowest binding energy of −10.1 and −9.8 kcal/mol with BACE-1 and γ-secretase, respectively. According to the above data, the ligand 6Z5 showed the lowest binding energy and was selected for further analysis as a possible inhibitor for BACE-1 and **γ-**secretase. Chemically the ligand 6Z5 is a pyrrolopyrimidinone compound having a phenylazanyl side-chain complexed with an indenyl group. The fused scaffold in pyrrolopyrimidine favors a more diverse and potent pharmacological profile. The heterocycles in pyrrolopyrimidine have demonstrated various biological activities, such as anti-cancer, antibacterial, antifungal, and anti-inflammatory effects [42]. Previous studies suggested pyrrolopyrimidinone as a possible inhibitor of microtubule-affinity regulating kinase and phosphodiesterase 5, potent AD drug targets [43–45].

Molecular dynamics simulation is a widely accepted method to explore the stability and dynamics of protein-ligand interactions [46]. Here, we have performed a 50 ns simulation on BACE-1-6Z5 and γ-secretase-6Z5 complexes to explore their dynamics, stability, and interaction pattern. The potential energies of BACE-1 and γ-secretase in their free, and their complex forms with 6Z5, were evaluated to observe the equilibration of systems. The average potential energies of free BACE-1 and BACE-1-6Z5 states determined by molecular dynamics simulation were found to be −3,271,200 and −3,271,386 kJ/mol, respectively. Similarly, the average potential energies of free γ-secretase and γ-secretase-6Z5 states were −1,921,257 and −1,921,301 kJ/mol, respectively.

**Figure 4.**
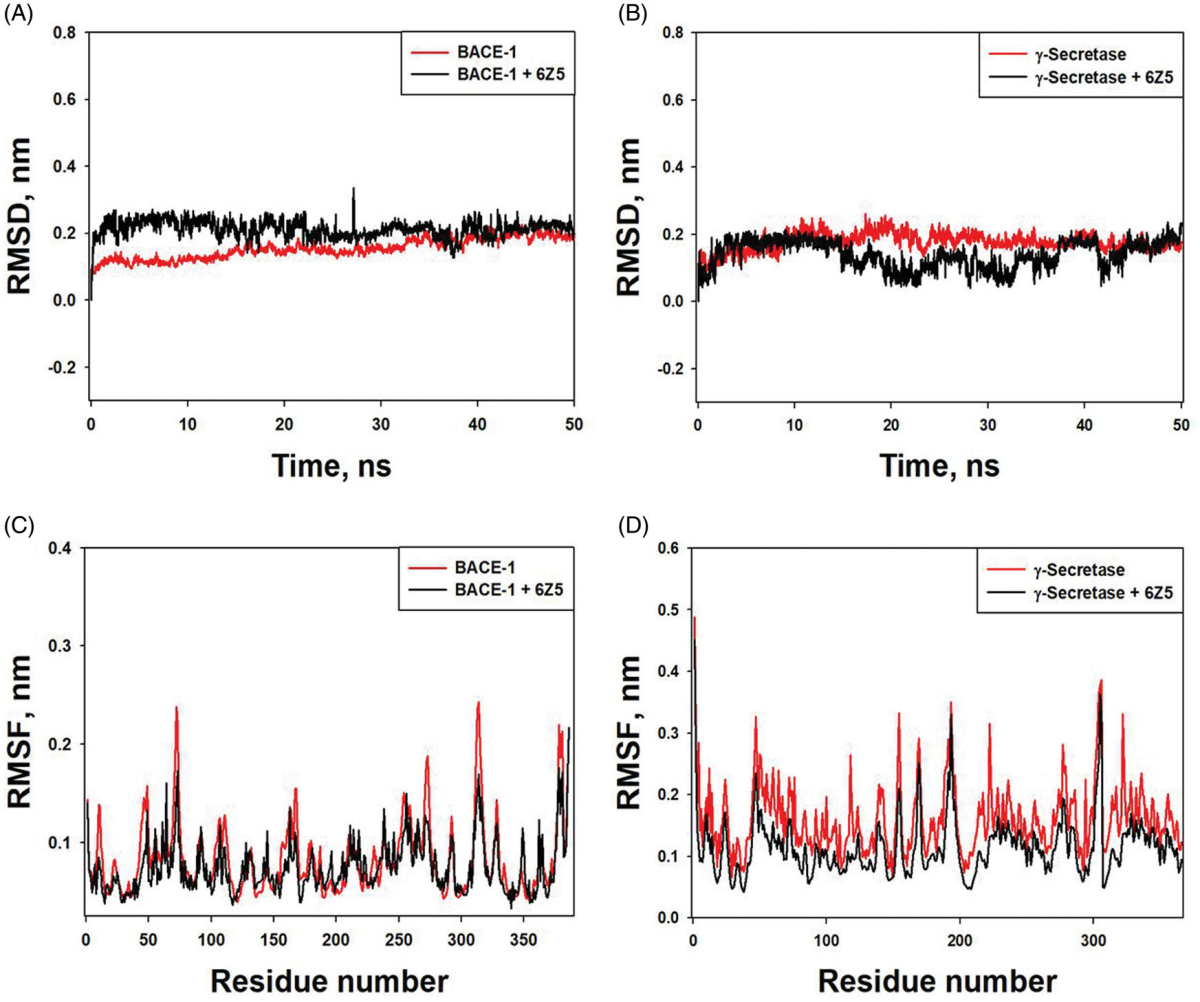
(A) RMSD *vs.* time plot for “free BACE-1” and “BACE-1-6Z5 complex”. (B) RMSD *vs.* time plot for “free γ-secretase” and “γ-secretase-6Z5 complex”. (C) RMSF *vs.* residue number plot for “free BACE-1” and “BACE-1-6Z5 complex”. (D) RMSF *vs.* residue number plot for “free γ-secretase” and “γ-secretase-6Z5 complex”.

In molecular dynamics, any alteration in the conformation of protein due to ligand binding can be measured by calculating the root mean square deviation (RMSD) [47]. In this study, the initial frames of BACE-1-6Z5 and γ-secretase-6Z5 were set as references, and the variability in RMSD of Cα-atoms was monitored. It was observed that the RMSD values of free BACE-1 and BACE-1-6Z5 complex varied between 0.0004–0.1825 and 0.0006–0.3253 nm, respectively (Figure 4(A)). Similarly, the RMSD values of free γ-secretase and γ-secretase-6Z5 complex were found to be in the range of 0.0005–0.2371 and 0.0004–0.2074 nm, respectively (Figure 4(B)). The average RMSD values of BACE-1 and γ-secretase alone were 0.1599 and 0.1769 nm, respectively, while RMSD values of BACE-1 and γ-secretase in their complex forms with 6Z5 were 0.2121 and 0.1398 nm, respectively. Since there were no significant variations in RMSD values of BACE-1 and γ-secretase due to the binding of 6Z5, it indicated that BACE-1-6Z5 and γ-secretase-6Z5 complexes were stable.

During molecular dynamics simulation, the variation in the conformation of amino acid residues is monitored by estimating root mean square fluctuation (RMSF) over the simulation time to gain an insight into the protein's overall conformational stability [48]. This study monitored the RMSFs of BACE-1 and γ-secretase in free forms and their complex structures with 6Z5 (Figure 4(C,D)). We observed some random fluctuations in different regions of BACE-1 and γ-secretase, which were minimized upon the binding of 6Z5. It was observed that the RMSF values of free BACE-1 and BACE-1-6Z5 complex varied between 0.0391–0.2434 and 0.0329–0.1757 nm, respectively (Figure 4(C)). Similarly, the RMSF values of free γ-secretase and γ-secretase-6Z5 complex were found to be in the range of 0.0653–0.3860 and 0.0424–0.3639 nm, respectively (Figure 4(D)). The average RMSF values of BACE-1 and γ-secretase alone were 0.0826 and 0.1601 nm, respectively, while RMSF values of BACE-1 and γ-secretase in their complex forms with 6Z5 were 0.0743 and 0.1141 nm, respectively. The overall results, therefore, suggest the formation of stable BACE-1-6Z5 and γ-secretase-6Z5 complexes.

**Figure 5.**
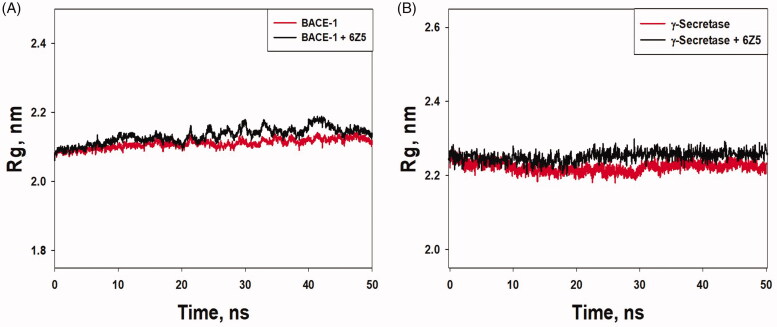
(A) Rg value *vs.* time plot for “free BACE-1” and “BACE-1-6Z5 complex”. (B) Rg value *vs.* time plot for “free γ-secretase” and “γ-secretase-6Z5 complex”.

**Figure 6.**
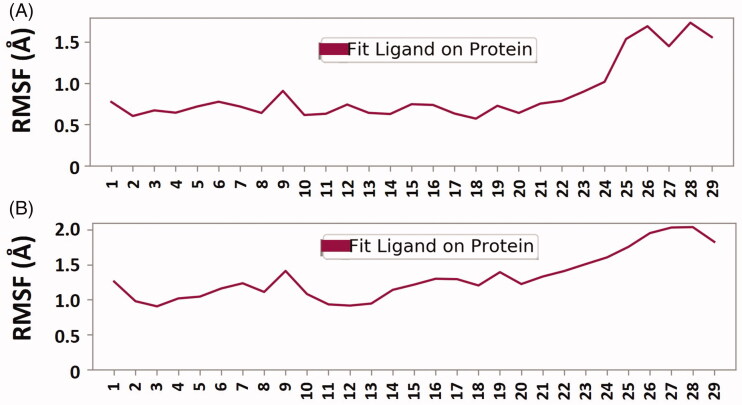
(A) Plot displaying ‘RMSF of ligand fit on protein’ for BACE-1. (B) Plot displaying “RMSF of ligand fit on protein” for γ-secretase.

The compactness, folding pattern, and conformation stability of a protein-ligand complex in different conditions can be estimated by observing the radius of gyration (Rg) as a function of simulation time [49]. Here, the Rg value of free BACE-1 and γ-secretase and their complexes with 6Z5 was monitored to evaluate the compactness of protein-ligand complexes (Figure 5(A,B)). The Rg values of BACE-1 alone and BACE-1-6Z5 complex were found to be 2.0991–2.1872 and 2.0892–2.1978 nm, respectively; while the Rg values of γ-secretase alone and γ-secretase-6Z5 complex were found to be in the range of 2.2007–2.2505 and 2.2260–2.3864 nm, respectively. The average values of Rg for BACE-1, γ-secretase, BACE-1-6Z5, and γ-secretase-6Z5 were estimated to be 2.1205, 2.2234, 2.1946, and 2.2682 nm, respectively (Figure 5(A,B)). The changes in Rg values of BACE-1 and γ-secretase upon the binding with 6Z5 were non-significant, thereby indicating the overall conformational stability of the complexes. The RMSF of ligand fit to respective proteins was also determined, as shown in Figure 6. It was evident that the RMSF values of 6Z5 did not fluctuate significantly (varied between 0.55 and 2.0 Å), implying a fine fitting of the ligand inside the binding pockets of BACE-1 (Figure 6(A)) and γ-secretase (Figure 6(B)).

**Figure 7.**
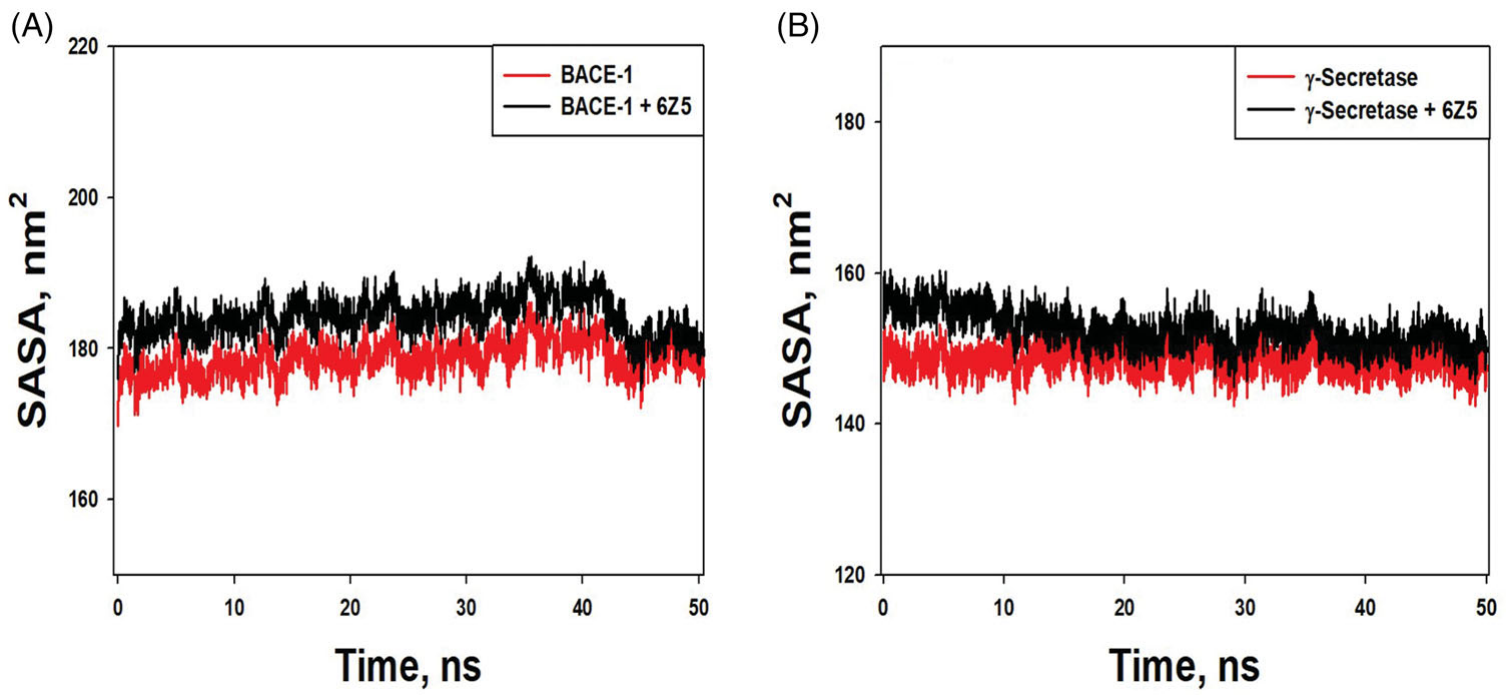
(A) SASA *vs.* time plot for “free BACE-1” and “BACE-1-6Z5 complex”. (B) SASA *vs.* time plot for “free γ-secretase” and “γ-secretase-6Z5 complex”.

**Figure 8.**
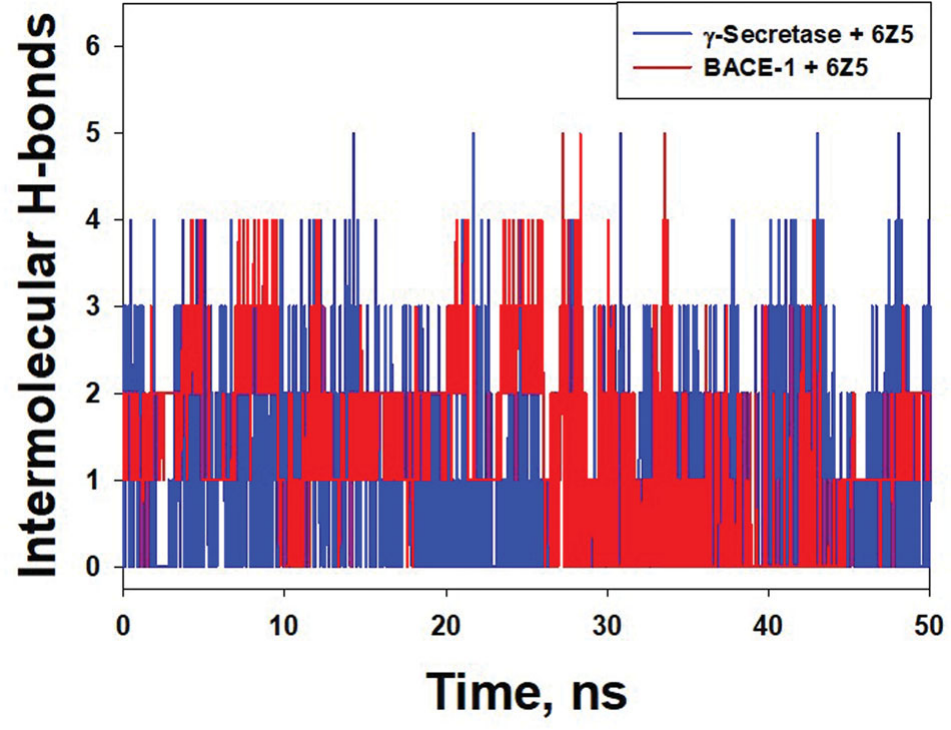
“Intermolecular H-bond” *vs.* time plot for “γ-secretase-6Z5 complex” and “BACE-1-6Z5 complex”.

**Figure 9.**
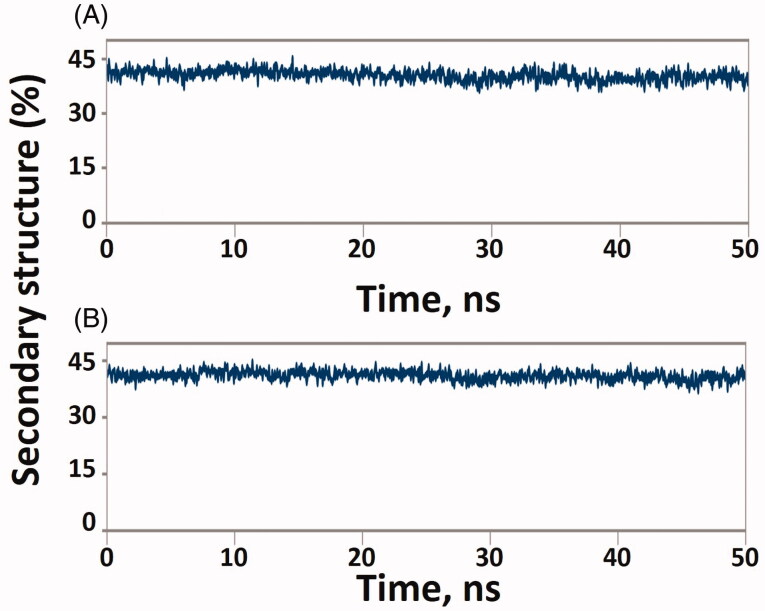
(A) Plot showing “% secondary structure of the BACE-1 protein in the presence of the 6Z5 ligand” as a function of simulation time. (B) Plot showing “% secondary structure of the γ-secretase protein in the presence of the 6Z5 ligand” as a function of simulation time.

Solvent accessible surface area (SASA) of a protein is defined as the exposure of the protein to the solvent. It is generally measured to understand a protein's folding pathway in altered conditions or ligand binding impact [50]. The SASA of BACE-1 and BACE-1-6Z5 fluctuated in the range of 174.97–108.51 and 176.86–188.82 nm^2^, respectively, while the SASA of γ-secretase alone and in γ-secretase-6Z5 complex form varied between 142.38–152.09 and 145.26–155.25 nm^2^, respectively (Figure 7(A,B)). The average values of SASA for BACE-1, γ-secretase, BACE-1-6Z5, and γ-secretase-6Z5 were found to be 180.26, 147.51, 188.82, 150.56 nm^2^, respectively. The results indicated no significant variations in SASA of BACE-1 and γ-secretase upon the binding with 6Z5. Further, the number of hydrogen bonds between proteins and ligands was determined (Figure 8). We found that the number of hydrogen bonds between BACE-1 and 6Z5 fluctuated between 0 and 5, with an average of ∼2.45 bonds. Similarly, the number of hydrogen bonds between γ-secretase and 6Z5 fluctuated between 0 and 5, with an average of ∼3 bonds. The stability of the protein-ligand complex was further evaluated by monitoring the secondary structure of a protein in the presence of a ligand (Figure 9). The percentage secondary structural of BACE-1 in the presence of 6Z5 was 40.43% (α-helix = 6.95% and β-sheets = 33.48%), while the percentage secondary structure of γ-secretase in the presence of 6Z5 was 41.02% (α-helix = 25.52% and β-sheets = 15.50%). All these parameters suggested the formation of a stable complex formed by the ligand 6Z5 with BACE-1 and γ-secretase.

The current study envisages the possible binding of protein targets BACE-1 and **γ-**secretase with active ligands with different structural orientations. Molecular dynamics simulations compensate for the shortcoming of molecular docking and consider the dynamics under physiological conditions as the process of ligand binding to the receptor. The 6Z5 was identified as one of the potential inhibitors by combining docking and MD simulation studies. Several studies have reported the binding free energies (predicted by the docking score of the protein-ligand complexes) to agree with the experimental binding affinities [51,52]. These predictive models also reflect the associated biological processes measured in molecular docking, virtual screening, and the ability of the ligand to bind to a specific receptor conformation [53]. To evaluate the quality of the predicted binding modes, we also calculated RMSDs between the predicted ligand binding modes and the released experimental complex structures. Despite the software result giving multiple docking conformation of protein/ligand binding affinity, we selected the lowest RMSD value as recommended earlier [54].

In contrast to studies on acetylcholinesterase inhibitors, the elucidation of AD therapeutic potential of BACE-1 and γ-secretase are inadequate because of the lack of potent and selective chemical probes [19]. These lacunae indicate the need to develop an efficient and specific inhibitor targeting these enzymes. Over the past two decades, pharmacological design and development of BACE-1 inhibitors with favorable physicochemical properties along with BBB permeability have undergone multiple challenging phases [55,56]. Ongoing experimental trials showed promising results of pharmacological BACE-1 inhibitors, MK8931, AZD‐3293, JNJ‐54861911, E2609, and CNP520, and are being intensively pursued as a therapeutic approach to treat AD patients [38]. Despite the high failure of lead drug candidates targeting BACE-1, this therapeutic strategy is not withdrawn, as it represents a pathologic mechanism-based treatment for AD. A recent study also encouraged novel compounds with an ultra-APP selectivity resulting in BACE-1 inhibitory effect [57]. Besides, BACE-1 inhibition has also been suggested as a combination therapy, a more effective way of improving cognition in AD [55]. In addition, γ-secretase inhibitors *viz.* avagacestat and semagacestat have undergone late-stage clinical trials for AD (phase II and phase III, respectively) [19]. However, these inhibitors have shown several side effects throughout the clinical trials [19].

The combined molecular docking and *in vitro* testing evaluated several compounds against BACE-1 and γ-secretase, as a safe target of Aβ reduction in AD therapy [58,59]. Despite their promising results, these compounds' failure rate in clinical trials is very high [19,38]. The failure during clinical trials upholds multi-target drug therapy, probably a better solution than focusing on a single target for complex neurological diseases like AD.

## Conclusion

The molecular docking studied ligands 55E, 6Z2, 6Z5, BRW, F1B, GVP, IQ6, and X37 showed good binding affinities towards two pharmacologically relevant enzyme targets of AD viz. BACE-1 and **γ-**secretase. These compounds showed favorable ADME properties and BBB permeation ability. Among these ligands, 6Z5 seems to be the most potent ligand which has shown multi-targeted binding affinity. However, the validations by *in-vitro* and *in-vivo* experiments are warranted. We believe that targeted modulation of BACE-1 and γ-secretase and other enzymes by our studied ligands especially, 6Z5, will be beneficial for the management of AD. Hence, we recommend the synthesis of this novel ligand that could target multiple enzymes involved in AD pathophysiology.

## Supplementary Material

Supplemental Material

## Data Availability

Data is available on request from the authors.
