# Supplementary material for: Concatenation of molecular docking and molecular simulation of BACE-1, γ-secretase targeted ligands: in pursuit of Alzheimer’s treatment
Source: Ann Med. 2021 Dec 10;53(1):2332–44. doi: 10.1080/07853890.2021.2009124 (PMC8667905; doi:10.1080/07853890.2021.2009124)
Supplement: Supplemental Material [file IANN_A_2009124_SM4163.docx]

**Supplementary Table: Structure of the Ligands**

| **No** | **Ligand ID** | **Name of the Ligands** | **Structure of the Ligands** |
| --- | --- | --- | --- |
| 1 | **55E** | 4-(4-hydroxy-3-methylphenyl)-6-phenylpyrimidin-2(5H)-one | 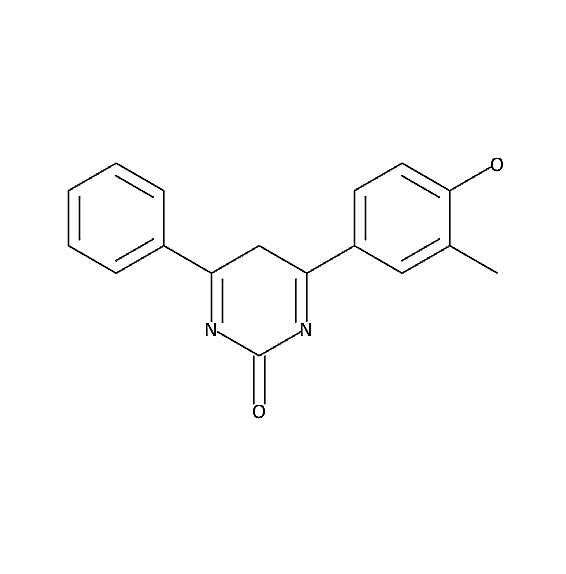 |
| 2 | **65A** | 4-(2-methoxyphenyl)-3,7,7-trimethyl-1,6,7,8-tetrahydro-5H-pyrazolo[3,4-b]quinolin-5-one | 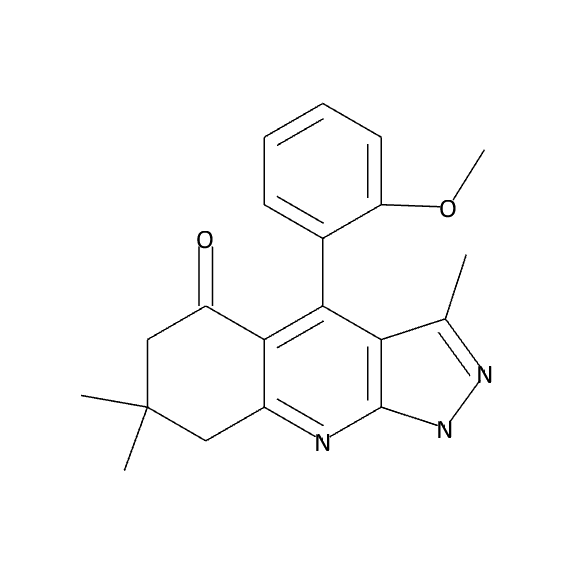 |
| 3 | **6VK** | (4~{S})-3-cyclopropyl-4,7,7-trimethyl-4-phenyl-2,6,8,9-tetrahydropyrazolo[3,4-b]quinolin-5-one | 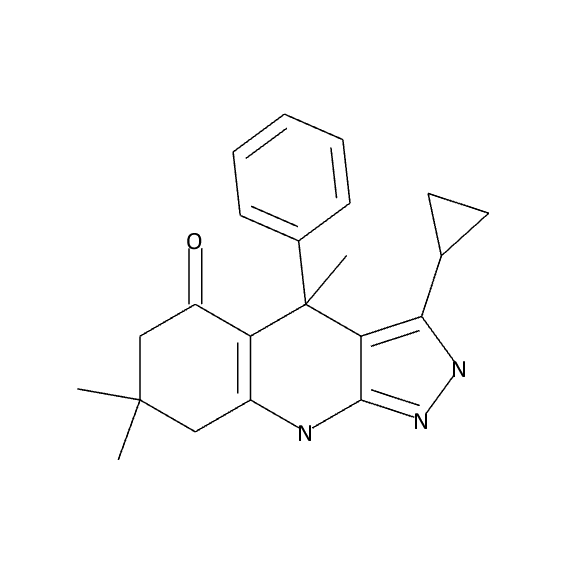 |
| 4 | **6VL** | (4~{S})-4-ethyl-7,7-dimethyl-4-phenyl-2,6,8,9-tetrahydropyrazolo[3,4-b]quinolin-5-one | 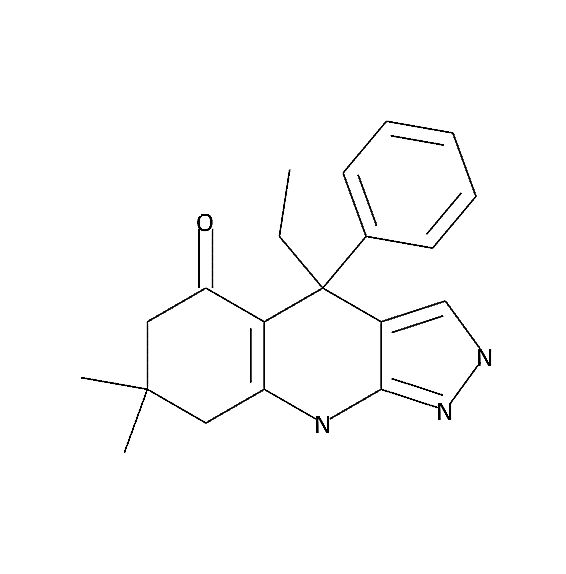 |
| 5 | **6VM** | (4~{S})-3-(2,2-dimethylpropyl)-4,7,7-trimethyl-4-phenyl-2,6,8,9-tetrahydropyrazolo[3,4-b]quinolin-5-one | 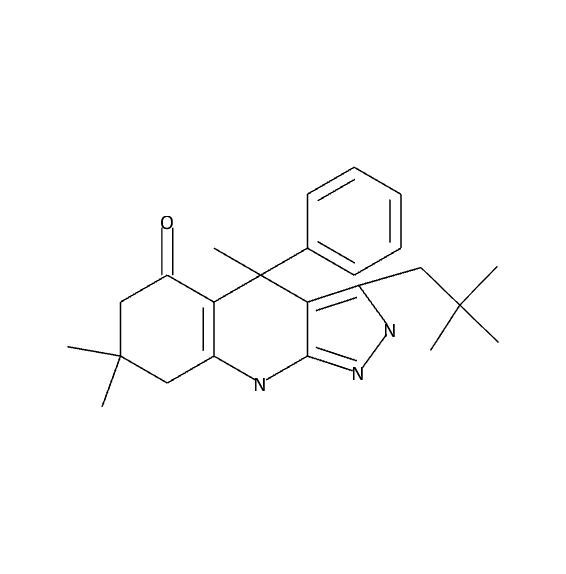 |
| 6 | **6Z2** | 7-[(1~{S})-1-(4-fluorophenyl)ethyl]-5,5-dimethyl-2-(pyridin-3-ylamino)pyrrolo[2,3-d]pyrimidin-6-one | 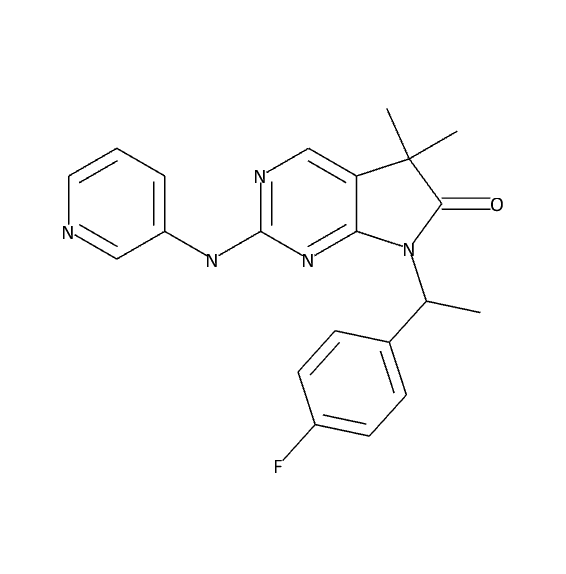 |
| 7 | **6Z5** | 5,5-dimethyl-7-[(1~{S})-4-oxidanyl-1~{H}-inden-1-yl]-2-phenylazanyl-pyrrolo[2,3-d]pyrimidin-6-one | 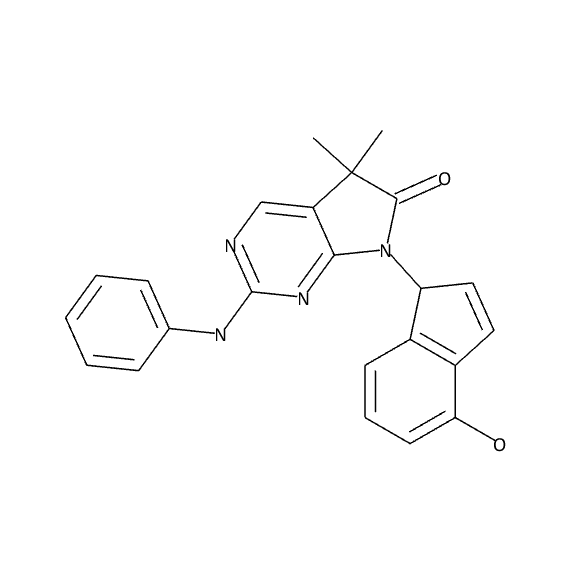 |
| 8 | **BRW** | *6-bromoindirubin-3'-oxime* | 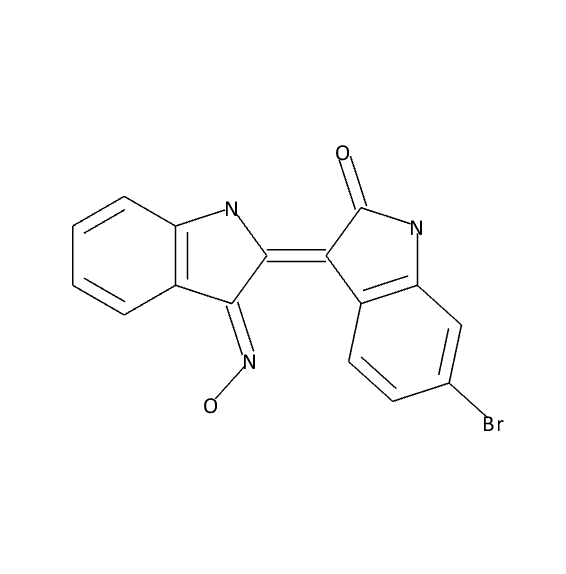 |
| 9 | **F1B** | (3~{Z})-5-ethanoyl-3-[[(1-methylpiperidin-4-yl)amino]-phenyl-methylidene]-1~{H}-indol-2-one | 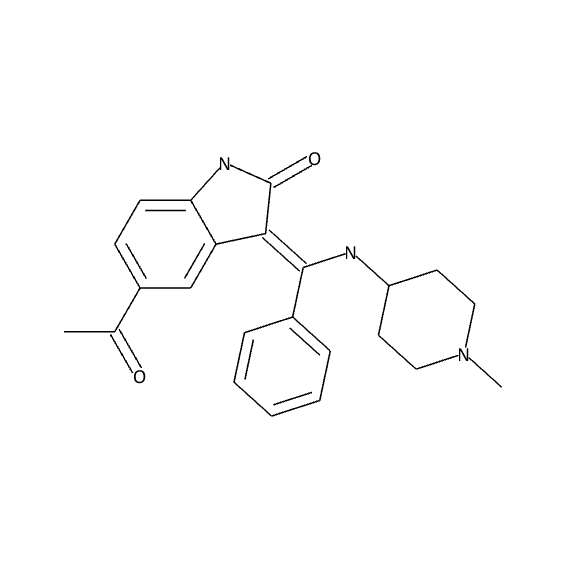 |
| 10 | **GVP** | 4-(4-Chlorophenyl)-4-[4-(1H-pyrazol-4-YL)phenyl]piperidine | 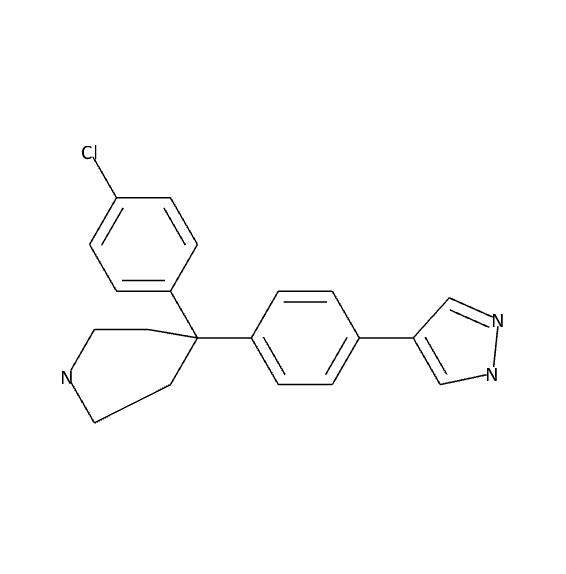 |
| 11 | **IQ6** | 6-chloro-N-cyclohexyl-4-(1H-pyrrolo[2,3-b]pyridin-3-yl)pyridin-2-amine | 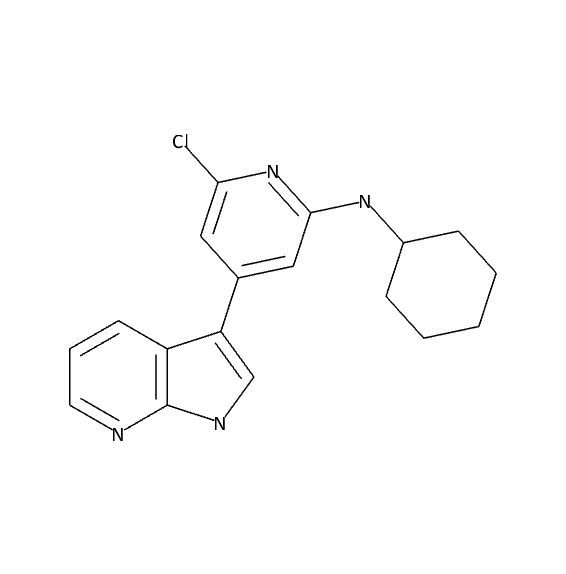 |
| 12 | **SMH** | N-(2-ethoxyethyl)-N-{(2S)-2-hydroxy-3-[(2R)-6-hydroxy-4-oxo-3,4-dihydro-1'H-spiro[chromene-2,3'-piperidin]-1'-yl]propyl}-2,6-dimethylbenzenesulfonamide | 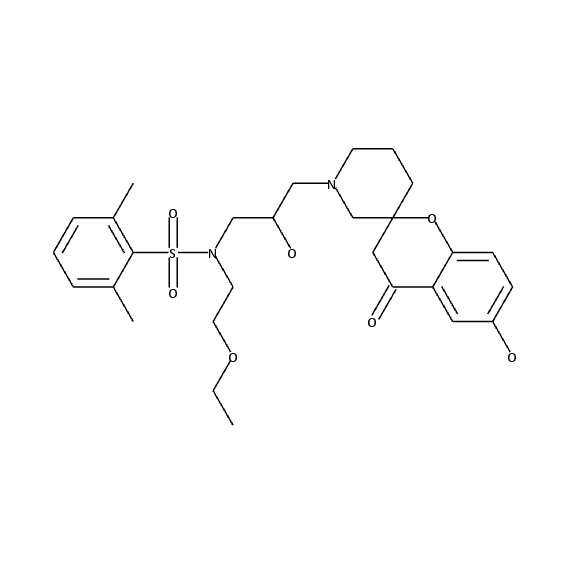 |
| 13 | **X37** | 4-(4-tert-butylbenzyl)-1-(7H-pyrrolo[2,3-d]pyrimidin-4-yl)piperidin-4-aminium | 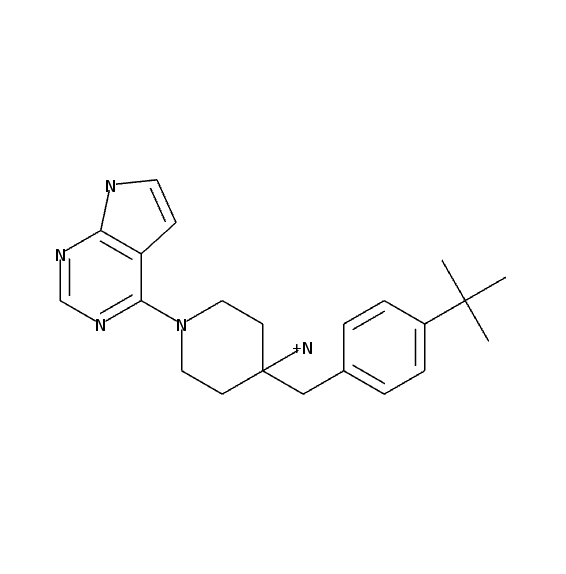 |
